# Morphological development of *Aspergillus niger *in submerged citric acid fermentation as a function of the spore inoculum level. Application of neural network and cluster analysis for characterization of mycelial morphology

**DOI:** 10.1186/1475-2859-5-3

**Published:** 2006-01-25

**Authors:** Maria Papagianni, Michael Mattey

**Affiliations:** 1 Department of Hygiene and Technology of Food of Animal Origin, School of Veterinary Medicine, Aristotle University of Thessaloniki, Thessaloniki 54006, Greece; 2 Department of Bioscience, University of Strathclyde, Royal College Building, 204 George street, Glasgow G1 1XW, UK

## Abstract

**Background:**

Although the citric acid fermentation by *Aspergillus niger *is one of the most important industrial microbial processes and various aspects of the fermentation appear in a very large number of publications since the 1950s, the effect of the spore inoculum level on fungal morphology is a rather neglected area. The aim of the presented investigations was to quantify the effects of changing spore inoculum level on the resulting mycelial morphology and to investigate the physiology that underlines the phenomena. Batch fermentations were carried out in a stirred tank bioreactor, which were inoculated directly with spores in concentrations ranging from 10^4 ^to 10^9 ^spores per ml. Morphological features, evaluated by digital image analysis, were classified using an artificial neural network (ANN), which considered four main object types: globular and elongated pellets, clumps and free mycelial trees. The significance of the particular morphological features and their combination was determined by cluster analysis.

**Results:**

Cell volume fraction analysis for the various inoculum levels tested revealed that by rising the spore inoculum level from 10^4 ^to 10^9 ^spores per ml, a clear transition from pelleted to dispersed forms occurs. Glucosamine formation and release by the mycelium appears to be related to spore inoculum level. Maximum concentrations detected in fermentations inoculated with 10^4 ^and 10^5 ^spores/ml, where pellets predominated. At much higher inoculum levels (10^8^, 10^9 ^spores/ml), lower dissolved oxygen levels during the early fermentation phase were associated with slower ammonium ions uptakes and significantly lower glucosamine concentrations while the mycelium developed in dispersed morphologies. A big increase in the main and total hyphal lengths and branching frequency was observed in mycelial trees as inoculum levels rise from 10^4 ^to 10^9 ^spores/ml, while in aggregated forms particle sizes and their compactness decreased.

**Conclusion:**

The methods used in this study, allowed for the detailed quantification of the transition between the two extreme morphological forms. The impact of spore inoculum level on the detailed characteristics of the particular morphological forms produced was high. Control of mycelial morphology is often regarded as a prerequisite to ensure increased productivities in industrial applications. The research described here demonstrates that adjusting the spore inoculum level controls effectively mycelial morphology.

## Background

In submerged culture the morphology of filamentous microorganisms varies between two extreme forms, pellets and free filaments, depending on culture conditions and the genotype of the strain. According to many reports, mycelial morphology is crucial to the process of fermentation, not only in relation to the shape of the hyphae themselves and the aggregation into microscopic clumps (micro-morphology), but also in the pelleted form of growth (macro-morphology). In all cases reported, the mycelium of acidogenic *Aspergillus niger *was found to conform to the morphological type described by Snell and Schweiger [1]: short, swollen filaments with swollen tips. The pellets should be small with a hard, smooth surface. It is known that this is brought about by adjustment of agitation and aeration, pH adjustment, concentration of important trace metals, and inoculum level [2].

Control of mycelial morphology in fermentations is often a prerequisite for industrial application. In some processes, free mycelia are required for increased productivities, as in the production of penicillin from *Penicillium chrysogenum *[3]. Whereas in other processes pellets or immobilized cells are required according to reports on itaconic [4] and citric acids [5], some fungal enzymes, such as polygalacturonidase or α-glucosidase [6], or steroid biotransformations [7]. Regarding pellet morphologies as a prerequisite for increased productivities, methodologies for pellet production for several microorganisms have been suggested [8,9]. However, reports on the preferred morphology are often contradictory since each one of the two extreme forms-pellets vs filaments- have their own characteristics concerning cell physiology, growth kinetics, nutrient consumption and broth rheology, which can be regarded either as advantages or as drawbacks.

An important drawback of pelleted suspensions is that cell growth- and consequent metabolic activity-occurs at the surface only, where contact with oxygen and medium nutrients is adequate, while the cells inside the pellets respond to a very different environment. Further into pellets, mass transfer limitation gradually occurs and cells become subjected to autolysis [10]. Obviously, only a fraction of the mycelium takes part in biosynthesis of metabolites when this requires for example elevated dissolved oxygen concentrations. There are cases however, where the drawback becomes certain advantage. Pellet formation results in striking effects on polygalacturonidase production by *A. niger *and its synthesis correlate well with the particular morphological type [6]. The more compact the pellet, the greater the enzyme synthesis. Regardless the medium used, an increase of almost two orders of magnitude in the polygalacturonidase concentration and production rates between the diffuse mycelium and pellets was observed. Similar increases were observed in α-galactosidase synthesis by mycelial pellets of *Mortierella vinacea *and nikkomycin production by *Streptomyces tendae *[11]. Such phenomena were related to diffusional limitations in pellets, which either reduce the extent of catabolic repression in pellets or limit the oxygen supply preventing thus an oxidative inactivation of specific sets of enzymes, as well as to the existence of additional factors, such as gradients of metabolic products in pellets serving as biological signals (modulators). In their review of the factors affecting mycelial morphology and metabolite production, Braun and Vecht-Lifshitz [11] suggest that mycelial aggregates may be viewed not merely as mechanical conglomerates, but rather as complex differentiated tissues, phenotypically characterized by specific metabolic activities.

There has been lots of discussion over the two extreme morphological forms, pellets and free filaments, since it has been early established that morphology affects the overall process productivities and subsequent economics. Between the two extreme forms can lie a wide range of morphologies, the existence of which is very often neglected, especially in rheological studies. A morphological form, which in the case of *P. chrysogenum *could account for over 90% of the biomass [12-14], is the form of mycelial clumps – aggregates, permanent in nature, which cannot be disintegrated by either dilution or gentle mixing. Such forms were never mentioned in early works on fungal morphology in submerged culture, due to the lack of adequate methods to monitor the morphology during fermentation. The application of image analysis systems in fungal biotechnology in the 1990s permitted the extraction of quantified information and the detailed characterization of various morphological forms. Using the image analysis method of Tucker et al. [14], Tucker and Thomas [15] described quantitatively the transition from pelleted to dispersed forms of growth of *P. chrysogenum *as inoculum levels rose towards 10^5 ^spores per ml of medium.

Research on the inoculum effects in commercially important fungal processes could now be more systematic, making use of the technological advances in monitoring and characterization of mycelial morphology during fermentation. Apart from the early works of Martin and Waters in 1952 [16], and Steel et al. in 1955 [8], which reported on some qualitative relationships, there are no reports in the literature on the effect of spore inoculum level in citric acid fermentation by *A. niger*. It is obviously a neglected area, since in terms of bulk production citric acid is widely regarded as one of the most important of the organic acids produced by microbiological methods. The aim of the presented investigations was to quantify the effects of changing spore inoculum level on the resulting mycelial morphology of *A. niger *in submerged culture and to investigate the physiology that underlines the phenomena. Fermentations carried out in a 3.0 L stirred tank bioreactor and inoculated with spore inocula in the range of 10^4 ^to 10^9 ^spores per ml of medium. Morphological features were evaluated by digital image processing and classified using an artificial neural network operating as an add-on to MS Excel. The significance of the various morphological features and their combination was determined by cluster analysis.

## Results

### Spore germination, hyphal branching and aggregation

**Figure 1:**
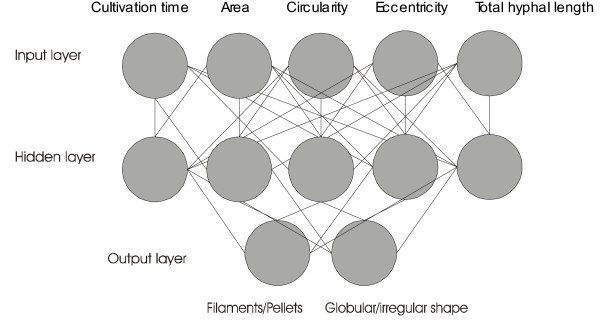
Structure of the artificial neural network (ANN). The combination of morphological features with main object types.

All runs were performed under the same reactor operating conditions while the only parameter that changed from run to run was the spore inoculum level which varied in the range from 10^4 ^to 10^9 ^spores/ml. Inoculation was carried out with non-agglomerated spores. Following inoculation, samples for morphological analysis and object classification by mean of the ANN (Fig. 1) were taken at 4, 8, 12, 14, 18 hours and then in regular intervals up to the maximum of 120 hours. No morphological changes were detected during the first 4 hours. At 8 hours, spores appeared swollen and a large fraction of them were agglomerated, while germination started and non-branched hyphae were formed. At 12 hours branching was apparent and at 18 hours permanent aggregates were detected.

### The citric acid fermentation

**Figure 2:**
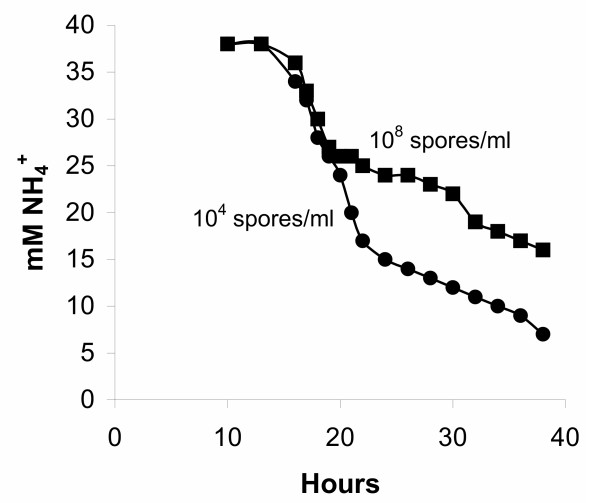
Ammonium ion levels in the broth of fermentations inoculated with 10^4 ^and 10^8 ^spores per ml.

In all fermentations, citric acid production commenced around 18 hours from inoculation. Citric acid production was high, exceeding in all experiments 100 g/l at 150 hours of fermentation. Citric acid production rate increased as the NH_4_^+ ^concentration in the fermentation medium fall to very low levels after the 36^th ^hour from inoculation. Biomass levels were comparable in all fermentations the first 72 hours (approximately, 3,5 g/l). Further in fermentation, those inoculated with spore inocula of the order of 10^7 ^and 10^8 ^spores/ml showed an increase in biomass accumulation and at 150 hours, time at which the runs were terminated, the final biomass concentration reached 9 g/l, while biomass concentration did not exceed 7 g/l in the cases of lower inocula. Inoculation with 10^9 ^spores/ml resulted in 8 g/l final biomass concentration. Dissolved oxygen levels in the bioreactor differed according to spore inocula concentrations. To concentrations above 10^6 ^spores/ml corresponding dissolved oxygen levels did not exceed an 80% of saturation at the 30^th ^hour point, while the corresponding levels for 10^4 ^and 10^5 ^spores/ml inocula were 100% saturated which remained such throughout fermentation. Dissolved oxygen concentration further decreased up to 70% during the course of fermentations inoculated with spore concentrations above 10^6 ^spores/ml. The (NH_4_)_2_SO_4 _uptake was calculated in all fermentations and found to be related to spore inoculum concentration, since cultures inoculated with spore concentrations exceeding 10^7 ^spores/ml showed a clear slower uptake (Fig. 2). Between approximately 36–40 hours, the bulk of ammonium was removed from the medium. The rate of uptake does not appear to have any relationship to the amount of biomass present in this stage, as biomass levels at 40 hours were comparable.

Glucosamine formation by the mycelium and subsequent release in the fermentation broth appeared to be related to spore inoculum level. Glucosamine detected in the fermentation broth at 18 hours in the cases of inocula 10^4^, 10^5^, and 10^6 ^spores/ml, while at 24 hours in all other cases where inoculum levels were higher. The maximum concentration of 46 g/l was noticed at 55 hours in fermentations inoculated with 10^4 ^and 10^5 ^spores/ml. This remained almost stable up to 90 hours of fermentation to drop sharply afterwards. Samples analyzed for glucosamine at the same timing showed that as the inoculum level increased to levels beyond 10^6 ^spores/ml, glucosamine accumulation decreased. When the inoculum of 10^9 ^spores/ml was applied, glucosamine levels detected at 55 and 70 hours were 21 and 18 g/l, respectively.

### The development of *Aspergillus niger *morphology

**Figure 3:**
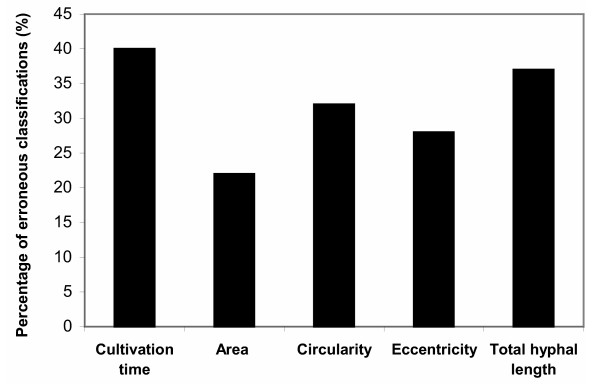
Significance of morphological features (probability of erroneous classification).

At 24 hours, depending on spore inoculum level, a morphological form trend was rather established-free filamentous or pellets for example, however it was still early for classification since the cultures were well in the growth phase and all kinds of morphological forms were detected. Specific growth rates were declined around 50 hours from inoculation while citric acid production rates increased (not shown). The comparison of the errors of the object identification my means of the ANN indicated that the best classification results were obtained in the period between 50 and 90 hrs in all cultures. The average of 70 hours was chosen for classification studies since morphology was established by that time in all cases and also because that time corresponds to the highest specific production rates observed (not shown). The significance (probability of erroneous classification) of the various morphological features of *Aspergillus niger *is shown in Fig. 3. According to the analysis, the area has the highest significance and the total hyphal length the lowest. The evaluation of the significance of the combination of these morphological features reveals that the combination of cultivation time with the object surface area and eccentricity yielded the highest significance or the lowest erroneous classification.

**Figure 4:**
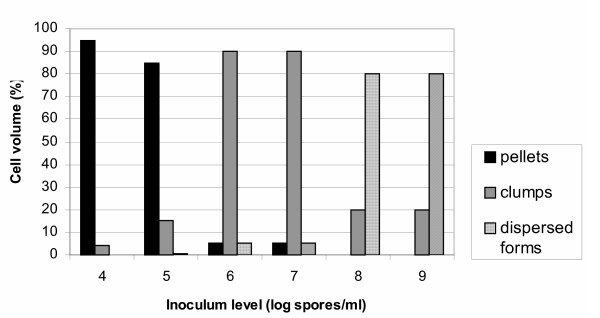
Cell volume fraction analysis in fermentations inoculated with spore inocula ranging from 10^4 ^to 10^9 ^spores/ml (70 hours).

Cell volume fraction analysis for the various inoculum levels is shown in Fig. 4. The figure shows the percentages of the various morphological forms at 70 hours cultures. The transition from pelleted forms to dispersed mycelium is clear as spore inoculum levels rise from 10^4 ^to 10^9 ^spores/ml.

**Figure 5:**
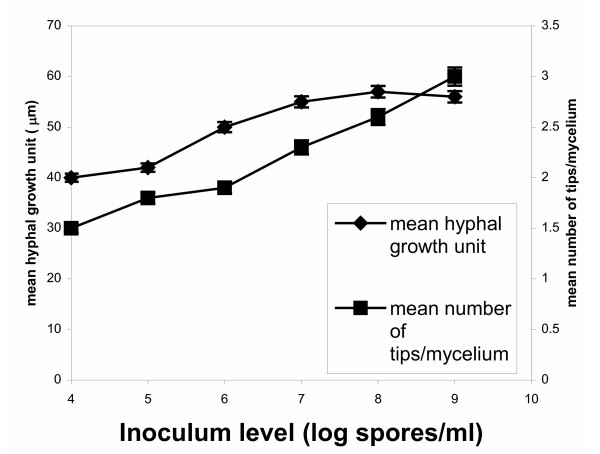
Effect of spore inoculum level on the dispersed morphological form. Mean hyphal growth unit and mean number of tips per mycelium at 70 hours of fermentations inoculated with spore inocula ranging from 10^4 ^to 10^9 ^spores/ml.

**Figure 6:**
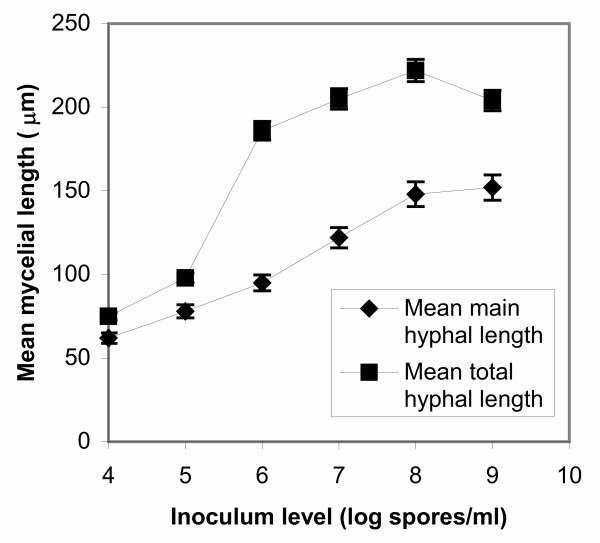
Effect of spore inoculum level on the dispersed morphological form. Mean main hyphal length and mean total hyphal length of mycelium at 70 hours of fermentations inoculated with spore inocula ranging from 10^4 ^to 10^9 ^spores/ml.

**Figure 7:**
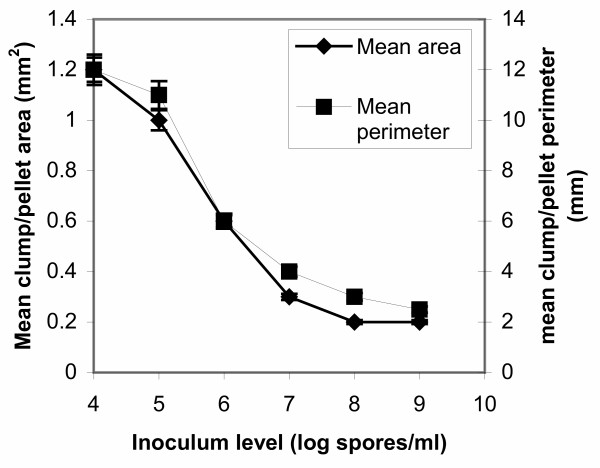
Effect of spore inoculum level on mycelial aggregation. Mean area and mean perimeter of pellets/clumps at 70 hours of fermentations inoculated with spore inocula ranging from 10^4 ^to 10^9 ^spores/ml.

**Figure 8:**
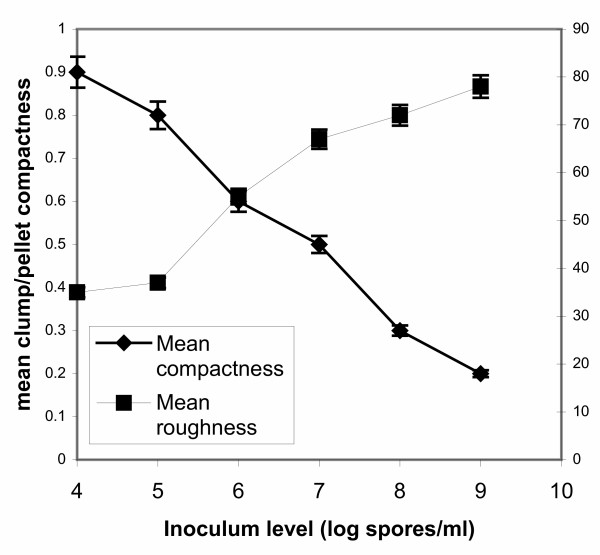
Effect of spore inoculum level on mycelial aggregation. Mean compactness and mean roughness of pellets/clumps at 70 hours of fermentations inoculated with spore inocula ranging from 10^4 ^to 10^9 ^spores/ml.

Since in all cases of inoculum levels various morphological forms were identified in samples apart from the dominating form, in figures 5, 6, 7, 8 all levels of tested inocula were plotted against morphological parameters corresponding to free filamentous or aggregated material. Figures 5 and 6 show the effect of spore inoculum level on the dispersed form, while Figs 7 and 8 show the effect on mycelial aggregation. As illustrated in these figures, variation of the inoculum level resulted in vastly different mycelial morphologies being developed by the end of the growth phase of a batch culture (70 hours). According to Figs 5 and 6, a big increase in the mean main hyphal length, mean total length and branching frequency of the mycelia was observed as the inoculum level increased from 10^4 ^to 10^8 ^spores/ml. A further increase from 10^8 ^to 10^9 ^spores/ml appeared to have only small additional effects mainly in the mean number of tips per mycelium. The aggregated form data showed similar behavior. As the inoculum level increased from 10^4 ^to 10^9 ^spores/ml there was a large decrease in aggregated particles size (Fig. 7) and a big change in compactness and roughness (Fig. 8).

## Discussion

Qualitative relationships between *A. niger *morphology and various process parameters in the citric acid fermentation like agitation, pH, and various medium constituents have been reported in many cases. Quantitative information however, became available with image analysis applications in more recent years. Most investigations on the interrelationship between mycelial morphology, metabolite production and process parameters concerned antibiotic fermentations. The citric acid fermentation by *A. niger*, regarding the relationship morphology/productivity, was the subject of comparatively a small number of reports [2]. As it appears in a number of reports, a considerable amount of work has been carried out on monitoring *A. niger *macro and micro-morphology during fermentations under various agitation levels [17,18] and mixing regimes [18], various glucose concentration levels in batch and fed-batch culture [19] and pH, phosphate and manganese concentration levels [20]. These works have been carried out with the same strain and reactor type, eliminating that way strain-related evaluation problems or reactor related process differences. In all cases, the system under investigation appeared to be very sensitive to changes in process parameters reacting morphologically and producing a wide array of morphologies, e.g. from filamentous to pelleted forms. Moreover, at increased stirrer rates (e.g. above 350 rpm), a prerequisite for achieving high citric acid yields, a cycle of mycelial fragmentation and re-growth has been observed and a transition from clumps to free filaments to clumps was monitored during the production phase, a situation that kept acid production rates at increased levels since the percentage of metabolically active mycelium maintained at increased levels through the formation of new branches from fragmented hyphae [21]. A similar pattern of fragmentation, re-extension, and further fragmentation has been reported in many cases for other filamentous microorganisms, grown under intensive agitation conditions [22-28].

In the case of the present work, detailed morphology investigations showed that after approximately 90 hours from inoculation, the pellet number (in pelleted fermentations) fraction passed a maximum, decreased and increased again. The same pattern was monitored with clumps. It was obviously the expected fragmentation and re-growth pattern. In filamentous broths, resulted from inocula of the order 10^8 ^and 10^9 ^spores/ml, fragmentation was indicated from variations in mean total mycelial lengths and mean lengths of zero-, first-, and second-order branches of mycelia. We have monitored mycelial morphology throughout fermentations in order to find the period that gives the most reliable quantitative information with respect to inoculum influence. Fragmentation and re-growth patterns were the subject of an earlier report on the same fermentation [21] and occur irrespective of the inoculum type. Therefore, in the present work we are reporting on the fermentation period in which morphology is established and it appears rather stable and this is before fragmentation and re-growth phenomena take place. According to preliminary studies on the errors of object identification, by means of the ANN, this period expands from 50 to 90 hours and as explained in the previous section, the figures given on morphological profiles refer to samples taken at 70 hours.

In most publications on citric acid fermentation by *A. niger*, the inoculum used was a preculture, grown in shake flasks (vegetative inoculum). This way, morphological development is predetermined to an extent and in any case, it strongly depends on the preculture's qualities. A strong-one of high biomass density-inoculum is always recommended for a successful fermentation. Inoculating directly with spores shifts fermentation backwards for a period corresponding to spore swelling, germination and branching. This initial fermentation period appears to be extremely important for the overall outcome in terms of morphology. Permanent aggregates (not detangled by shaking) were detected at 18 hours in all fermentations, while at 24 hours a morphological trend was established-pellets or free filamentous forms- and it depended on the spore inoculum level.

There has been some discussion in the past regarding the factors affecting mycelial aggregation and pellet formation in filamentous microorganisms. In their review, Braun and Vecht-Lifshitz [11] listed a number of factors that influence pellet formation as microbiological (genetic, cell-wall composition, inoculum size, growth rate, nutrition, carbon: nitrogen ratio) and physicochemical (shear forces, surface-active agents, pH, temperature, Ca^2+ ^ions, ionic strength, suspended solids). However, assessing these factors even for the familiar industrially important *Aspergillus *and *Penicillium *species leads to contradictory reports [3-5,29]. Despite the contradictions, the effect of various fermentation parameters on pellet formation seems to be quite similar in filamentous systems as genetically remote as fungi and actinomycetes. Thus, in *P. chrysogenum *[30], *A. niger *[31,32], *Streptomyces tendae *[33], and *S. griseus *[11], pellets are formed at inoculum below 10^11 ^spores/m^3 ^(fermenter volume), while at higher inocula filamentous growth prevails. Also, factors favoring increased growth rates, such as media rich in easily assimilable nutrients, have been discussed in some cases to reduce pellet formation in fungi [30] and actinomycetes [33]. Such observations led to a limitation hypothesis [11] which suggested that the lack of any particular nutrient, including oxygen, induces pellet formation. Increased mycelial aggregation was noted as a consequence of nutrient limitation, especially nitrogen [6,33], although with respect to oxygen reports are contradictory. From existing reports it is difficult to draw a conclusion about oxygen since no direct comparisons with various oxygen levels were made. Concerning the common observation [4,5,30] of predominating pelleted morphologies in the early life of a culture (sufficient oxygen supply) turning filamentous in later stages (oxygen limited cultures), this is rather a result of increased vacuolation and subsequent mycelial fragmentation and re-growth under conditions far different from those on the onset of fermentation.

In the present work, trap-net formation and stabilization of mycelial aggregates in fermentations inoculated with 10^4^, 10^5 ^and 10^6 ^spores/ml corresponded to increased dissolved oxygen levels, a fact that is opposite to the above mentioned limitation hypothesis regarding oxygen. More interesting is the observation that a faster (NH_4_)_2_SO_4 _uptake was recorded in these fermentations (Fig. 2), followed by formation and release of glucosamine in earlier time (detected at 18 hours in the broth) and at elevated amounts compared to fermentations inoculated with higher spores concentrations. In our recent work on the fate and role of ammonium ions during the citric acid fermentation by *A. niger *[34], it has been shown that glucosamine is the product of the relationship between glucose and ammonium during the early stages of the citric acid fermentation process by *A. niger*. That study focused on the early fermentation stages and in particular on the fate and role of ammonium ions and the overall dynamics of the system under production conditions. By applying the optimum initial ammonium ions concentration in the medium (the same amount of (NH_4_)_2_SO_4 _applied in the present study), this has to be depleted before citric acid production establishes. The bulk of ammonium is removed from the broth between 20 and 25 hours and it is almost depleted by 36 hours. The uptake of ammonium ions is followed by a release of protons. Detailed time studies on the relationship between ammonium ion uptake and proton release carried out by Wayman [35] showed that the two are linked but only indirectly. The release of protons into the broth does not coincide precisely with the uptake rate of ammonium ions, but lags a couple of hours. This delay precludes a proton/ammonium antiport as the means of ammonium uptake. Coincidently, peaks in the rate of ammonium uptake follow peaks in the growth rate by about 4 hours. A chain of events where growth leads to ammonium uptake leads to proton release is established in the early phase. However, one phenomenon that does coincide precisely with ammonium uptake is glucose uptake which is at such a high level (150 g/l) that it must be protein mediated.

As presented in the results section, the bulk of ammonium was removed from the medium between approximately 36–40 hours, while maximum glucosamine concentrations in fermentations inoculated with 10^4 ^and 10^5 ^spores per ml, reached 46 g/l at 55 hours. Comparing these results with those reported in our previous publication [34] we observe a significant delay in the process of glucosamine formation and release which is obviously attributed to the type of inoculum (spores vs vegetative inoculum). Also, both (NH_4_)_2_SO_4 _uptake and glucosamine concentrations in the broth appear to be related to the spore inoculum concentration. Mycelial aggregation is enhanced under conditions that ensure increased dissolved oxygen levels (e.g. spore inoculum concentration up to 10^6 ^spores per ml). Under such conditions, ammonium ion uptake is faster followed by a faster formation and release of glucosamine, which certainly contributes, as a sticky substance, to the development of permanent aggregates. The significantly lower glucosamine levels in fermentations initiated with stronger spore inocula correspond to filamentous morphological forms.

The role of aeration in citric acid fermentation by *A. niger *is well known (10). Low aeration levels result in lower productivities. Several aspects of the role of aeration and dissolved oxygen have been investigated so far [36]. However, the relationship between dissolved oxygen levels in the bioreactor and the development of fungal morphology is not yet fully understood [10]. Here, a relationship between dissolved oxygen levels and ammonium ion uptake, which reflects on the biosynthesis of glucosamine, seems to exist. The rate of uptake does not have any relationship to the amount of biomass present at this stage, as biomass levels at 40 hours were comparable. Obviously, this relationship influences the development of fungal morphology since no other parameter appears to have an effect in this set of experiments. There are no reports in the literature regarding dissolved oxygen concentration and ammonium ions uptake. It is well known that citric acid production commences with the depletion of ammonium in the broth [36]. It is also well known that high dissolved oxygen levels are associated with high citric acid production rates [37]. It is expected therefore, that whatever causes a delay in the transfer of ammonium ions inside the cell will cause a delay in citric acid production rate. Low oxygen concentrations in the broth may interfere to the proton/ammonium antiport [34] resulting in lower ammonium ions uptake rates and lower citric acid production rates. The fast process of glucosamine accumulation [34] under such conditions is also affected. The concentration of the polysaccharide on the surface of the hyphae is expected to play a role in the formation of permanent aggregates due to the nature of the compound, which acts as a surface adhesion agent. Lower dissolved oxygen concentrations in our case resulted from increased inoculum levels. The relationship between dissolved oxygen and ammonium ions uptake, irrespective of the spore inoculum level, needs further investigation which is beyond the aim of the present report.

In their review on mycelial morphology and metabolite production, Braun and Vecht-Lifshitz [11] wrote that: "surface modification affects the aggregation directly. However, it is difficult to assign some factors (e.g. growth rate, limiting nutrients or inoculum size) as being responsible specifically for cohesion or disintegration of mycelial aggregates". Our results show that inoculum size determines fungal morphology indirectly by influencing the environment inside the bioreactor and consequently fermentation rates, affecting thus polysaccharide formation which at the onset of fermentation appears to be critical for the development of particular morphological forms.

Application of neural network and cluster analysis for characterization of fungal morphology has been reported so far only by Gerlach et al. [12]. In that report, the influence of temperature, phosphate concentration, and agitation was investigated on the morphology of *A. awamori *in shake flasks cultures and airlift bioreactors and the relationship of fungal morphology to process performance were discussed. Our results on the significance (probability of erroneous classification) of the various morphological features of *A. niger*, as shown in Fig. 3, show that the area has the highest significance while the total hyphal length the lowest. Results of the same trend were reported by Gerlach [12] for *A. awamori*, who also found that the combination of cultivation time with the object surface area and eccentricity yielded the highest significance. Unfortunately, in that study a vegetative inoculum (preculture) was used for bioreactor experiments eliminating that way any inoculum effect.

As illustrated in Figs 4, 5, 6, 7, and 8, varying the spore inoculum level resulted in vastly different mycelial morphology being developed by the end of growth phase of batch cultures. Fig. 4 shows the cell volume fraction analysis (70 hours) for the various spore inoculum levels applied in this study. A clear transition is shown to take place from pelleted to dispersed forms as inoculum levels rise from 10^4 ^to 10^9 ^spores/ml. Inoculating with 10^4 ^spores/ml resulted in pellets that accounted for the 95% of the detected objects. The 10^5 ^spores/ml inoculum gave a mixture of pellets and clumps, with the pellets predominating and the clumps accounting for a 15%. There was not any significant difference for inocula of 10^6 ^and 10^7^spores/ml. In both fermentations, clumps accounted for the 90% of detected objects. Inoculation with 10^8 ^and 10^9 ^spores/ml resulted in dispersed mycelium (free filamentous form) and a small proportion (20%) of clumped mycelium. Quantitative characterization of *A. niger *morphology in the citric acid fermentation as a function of the spore inoculum level is being reported here for the first time.

As mentioned in the introduction, the majority of the published studies involving image analysis deal with the morphology of *Penicillium *species or commercially important actinomycetes. Fraction analysis of the various morphological forms of *Streptomyces clavuligerus *and *P. chysogenum *in submerged fermentation has been done by Tucker et al. [14] in testing a method for fully automatic image analysis of mycelial morphology. The method allowed rapid measurements of important morphological parameters of the freely dispersed mycelia but most importantly it provided a novel characterization of the clumped form which constitutes more than 90% of the biomass in some fermentations (as in the present case) and might therefore be expected to have a major influence on broth rheology, fermenter mixing, mass transfer, and hence fermentation productivity. Based on object detection methods similar to those of Packer et al. [38] and Paul et al. [39], Gerlach et al. [12] reported on cell volume fraction analysis in submerged cultures of *A. awamori *studying the influence of reactor systems on fungal morphology. In these cases the effect of the spore inoculum level was not among the subjects of the investigations.

The effect of the spore inoculum level on fungal morphology was the subject of the report by Tucker and Thomas [15] with *P. chrysogenum*, who used image analysis to show a sharp transition from pelleted to dispersed forms as the spore inoculum increased from 5 × 10^4 ^to 5 × 10^5 ^spores/ml. Although the applied inoculum level limits were narrow and the study was carried out in shake flasks, the reported results are sound and in general agreement with previous observations (qualitative) on the same microorganism by Smith and Calam [40]. The influence of initial spore concentration on agglomeration leading to pellet formation in *P. chrysogenum *cultures was also studied by Nielsen et al. [25]. For a low concentration of spores in the inoculum, the authors found that only a few elements agglomerate and pellets with a small diameter were obtained. At higher spore concentrations, many hyphal elements agglomerated and developed into large diameter pellets. In that study, spore concentrations used for inoculation were in the order of 10^7 ^spores/l (from 3.5 × 10^7 ^to 8.6 × 10^7 ^spores/l), and although image analysis was used to quantify morphology in that work, no distinction was made between pellets and clumps, which in other works with the same microorganism [8] appear to account for more than 90% of the detected mycelial particles.

As explained in the Results section, in figures 5, 6, 7, 8 all levels of tested inocula were plotted against morphological parameters corresponding to free filamentous or aggregated material because in every single experiment, apart from the dominating form, various morphological forms are identified. According to these figures, the impact of the spore inoculum level on the detailed characteristics of a particular morphological form is high. A big increase in the mean main hyphal length, total hyphal length, and branching frequency of the mycelium is observed as inoculum level rises from 10^4 ^to 10^8 ^spores/ml, and only a small additional effect in the mean number of tips per mycelium is observed at 10^9 ^spores/ml (Figs 5, 6). This means that in the case of fermentations in which entangled mycelium predominates, the free filamentous material is of small size, which is logical and it might result from fragmented hyphae or from spores that germinated later than others. The aggregated form gave similar results. Rising the inoculum from 10^4 ^to 10^9 ^spores/ml, the size of the detected aggregated particles decreases, their roughness increases and the compactness decreases (Figs 7, 8). Results of a similar trend were reported by Tucker and Thomas [15] for *P. chrysogenum*: increasing the spore inoculum level from 5 × 10^4 ^to 5 × 10^5 ^spores/ml caused a large decrease in clump size and a big change in compactness and roughness. Further increasing the spore inoculum level decreased slowly the percentage of clumps. This is directly opposite with the observations of Nielsen et al. [25] again for *P. chrysogenum *who found that pellet diameter increased with spore concentration. Nielsen et al. [25] discussed the different behavior of their microorganism from that of *Aspergillus *as it appears in the work of Takahashi and Yamada [31,32] and explained it on the basis of the different mechanism of spore coagulation in *Aspergillus *and *Penicillium *as proposed by Takahashi and Yamada (36): The spores of *Aspergillus *are of the coagulative type (spores coagulate whilst germinating and give rise to a net of intertwining hyphae), while the spores of *Penicillium *are of the non-coagulative type (a single spore is able to develop into a pellet). This mechanism does not seem to apply in our case, as in many others [9,25], and the use of image analysis systems in studies of fungal morphology made it clear that agglomeration is determined not only by the organism but also by the environmental conditions. Obviously, direct comparisons of works carried out with different organisms (even different strains of the same organism), media and bioreactors are not feasible. Nielsen et al. [25] commented on such contradictory observations as follows: "Thus, on the basis of our present experimental data, it is concluded that agglomeration leading to pellet formation is not simply determined by the probability of physical contact between hyphal elements".

Changes in fractions of the various morphological types or in characteristics of aggregated and dispersed forms that may occur further in fermentation were beyond the aim of the present study. Also, beyond the aim of the present study was to relate the changes observed with final yields of citric acid. However, as the present results suggest that fungal morphology depends strongly on spore inoculum level, it may be possible to manipulate it in order to avoid morphologies like e.g. the freely dispersed form, which in high biomass concentrations would cause serious rheological problems and low productivities. Considering the results reported in the present study, it must be stressed that these apply to citric acid producing *A. niger*. Different medium formulations may affect spore germination. Spore stock preparation conditions are also very important. And, although similar morphological forms in various filamentous microorganisms may be produced under similar controls, it is better to avoid the treatment of these results as a general phenomenon. It is very characteristic that only a small increase (from 5 × 10^4 ^to 5 × 10^5 ^spores/ml) in *P. chrysogenum *spore concentration was required in the report of Tucker and Thomas [15] to produce a big and sudden change in morphology, from pelleted to filamentous. In the case of the present report, *A. niger *spore inoculum concentration should be increased by many orders of magnitude (from 10^4 ^to 10^8^) to produce similar results.

Image analysis produces a large number of object features, which vary widely during fermentation. These features however, do not yield by themselves sufficient information about the morphological state. Their combination, including the fermentation time, by means of an artificial neural network, permits their classification into general object types. Cluster analysis allows the evaluation of the significance of the object features and their optimal combination for the entire fermentation type. The present research revealed that the combination of only a few object features has much higher significance than that of all of the features and this is in agreement with the results presented by Gerlach et al. [12]. The methods used in this study, allowed for the detailed quantification of the transition between the two extreme morphological forms. It would be valuable therefore, in investigations on the effect of culture conditions on the early progress of spore inoculated fermentations.

## Conclusion

The aim of the present work was to quantify the effect of spore inoculum level on fungal morphology of *A. niger *in the commercially important fermentation of citric acid. This was achieved by using image analysis in combination with an artificial neural network and cluster analysis. The methods used in this study, permitted the detailed quantification of the transition between the extreme morphological forms of pellets and free filaments. The impact of spore inoculum level on the characteristics of each particular morphological form produced by changing the concentration of spores in the inoculum was high. Control of mycelial morphology is often regarded as a prerequisite for industrial application. The research described here demonstrates that adjusting the spore inoculum level controls effectively mycelial morphology.

## Materials and methods

### Material

Chemical compounds used in this study were purchased from SIGMA-ALDRICH fine chemicals (Missouri, U.S.A.) and Oxoid (Basingstoke, U.K.). Filters used for cell dry-weight measurements were by Whatman International (Maidstone, U.K.).

### Microorganism, inoculum preparation, medium

An industrial strain of *Aspergillus niger *(*A. niger *PM1, University of Srathclyde) was used throughout this work. This was maintained on molasses agar, which contained 300 g/l cane molasses (pH adjusted at 6.8), and 18 g/l agar (Technical, Grade 3, Oxoid). The plates were incubated at 30°C for 7 days. Spores were collected from mature culture plates and spore suspensions were diluted with sterile medium to make a range of concentrations in the order of 10^4 ^spores/ml to 10^9 ^spores/ml of media.

The composition of the fermentation medium was the following (g/l): D-Glucose, 150; (NH_4_)_2_SO_4_, 2.5; MgSO4·7H_2_O, 0.5; KH_2_PO_4_, 2.0; Fe^3+ ^[as Fe_2_(SO)_4_·24H_2_O], 0.1 × 10^-3^; Zn^2+ ^[as ZnSO_4_·7H_2_O], 0.1 × 10^-3^; Cu^2+ ^[as CuSO_4_·5H_2_O], 0.06 × 10^-3^.

### Culture conditions

The stirred tank bioreactor used in this work was a 3.0 L New Brunswick Scientific BIOFLO 110. The reactor was equipped with baffles. The agitation system consisted of two 6-bladded Rushton-type impellers (52 mm), operating at a stirrer speed of 400 rpm. Process temperature was maintained at 28°C and the airflow rate at 1 vol/vol/min air/medium (vvm). pH was controlled at 2.1 by the automatic addition of titrants (2 M NaOH and 20% H_2_SO_4 _solutions). Polyethylene glycol (M.W. 2000, SIGMA) was used as antifoam in all fermentations. Fermentations terminated at 150 hours from inoculation.

### Analytical methods

Dry weights were determined by filtering 20 ml of broth through pre-weighed glass fiber filters (grade GF/C, 4.25 cm, Whatman), washing and drying in a microwave oven (15 min at low power) and left in a dessicator for 24 hours before reweighing. Citric acid was determined by the method of Marier and Boulet [41]. Glucose was determined by using a glucose oxidase/peroxidase method as described by Kunst et al. [42].

The concentration of ammonium ions in solution was calculated using an ammonium electrode (Asea Brown Boveri/Kent Taylor 8002-8). A protocol was developed [35] to allow for good reproducibility over a large volume of samples and an extended period of time. Concentrations of ammonium ions within the mycelium were calculated by filtering the broth and washing the filter cake with tap water (buffered to the same pH as the broth with HCl). The cells were then re-suspended in a small quantity of buffer and the cell membranes were disrupted by further addition of methanol to make a suspending solution of approximately 50% strength. After standing for 24 hours and removal of the solids, the ions were measured with the electrode.

Glucosamine determination was done by HPLC analysis (using a mass detector). The concentration range of standards (provided by SIGMA, D(+)-Glucosamine Hydrochloride, C_6_H_13_NO_5_-HCl, MW 215.6) was 30, 15, 7.5 and 3.75 mM and they were assayed using a Redex RNM Carbohydrate column (Phenomenex, 300 × 7.8 mm) and a mass detector (Sedex 55). The standards used for glucose were 54, 27, 13.5, and 5.4 mM. The mobile phase was water, the flow rate 0.4 ml/min (0.6 ml/min for glucosamine). The procedure was performed at 85°C and a pressure of 1.9 bars and repeated three times. The software used was the Gilson 715 HPLC.

### Image analysis and processing

Fungal morphology was characterized by using a semi-automatic image analysis system consisting of an Olympus microscope (Olympus, New Hyde Park, NY, U.S.A.) operated as phase contrast, a CCD camera (Sony, Cambridge, U.K.), a PC with a frame-grabber, and the image analysis software (SIS, Olympus, Germany). Samples preparation and measurements were as described in earlier publications [18,19,43]. A magnification of 100× was applied for measurements on mycelial particles. For the individual mycelia, the area and perimeter measurements were used to estimate other morphological parameters. These included the length of the longest connected path through the mycelium (main length); the total length of all the hyphae in the mycelium (total length of the skeletonized image); the number of tips; and the hyphal growth unit (total length divided by the number of growing tips). For all detected objects, the following features were evaluated: area, circularity, eccentricity, total hyphal length, and branching frequency. The eccentricity was determined according to Pitas [44]. Classification of detected objects was done by means of an artificial neural network operating as an add-on to MS Excel. Four object classes were defined: filamentous mycelium (-1/-1), clumps (-1/1), globular pellets (1/1) and elongated pellets (1/-1). The numbers in brackets are the codes used for the training of the neural network. The training of the neural network was performed by back-propagation, recommended for feed-forward nets by Rumelhart et al. [45].

To evaluate the significance on the classification decision of the neural network of the various morphological parameters, cluster analysis was applied as described by Gerlach et al. [12]. Cluster analysis considers a multidimensional space of state consisting of normalized data. The ranges of the data of the target function were first decomposed into clusters of the same size. Cluster centers were then formed from the data in each of the clusters and the distance of the measured values of the respective parameter from the cluster center was calculated according to geometrical criteria. The classification was characterized as correct when the distances of the respective measured data from their center were smaller than their distances from any of the other cluster centers. When this was not the case, classification was erroneous, with a low number of erroneous classifications indicating a close correlation of the respective parameter to the target function.

Clumps and pellets were characterized in terms of area, perimeter, compactness and roughness. Compactness was estimated by the ratio of the area of the hyphae in the clump/pellet to the total area enclosed by its actual outer perimeter [14]. Roughness is given by the circularity factor. The percentage of mycelia in the forms of clumps and pellets was estimated according to Tucker et al. [14]. The fungus grows in spherical or elongated particles which are distinguished between pellets and clumps or as individual mycelial trees. Depending on the result of process morphological monitoring, the volume of the cells was calculated from the projected surface area of the objects according to Packer et al. [38] and Paul et al. [26].

Fermentations were carried out in triplicates. For morphology measurements an average of 500 objects were measured per sample. Morphological data are presented as mean values.

## Authors' contributions

Both authors contributed equally in this work
